# *ParPMC*-mediated susceptibility to plum pox virus: vascular expression in *Prunus armeniaca* and functional validation through ortholog silencing in *Nicotiana benthamiana*

**DOI:** 10.3389/fpls.2025.1614211

**Published:** 2025-06-25

**Authors:** Ángela Polo-Oltra, Jesús A. Sánchez-Navarro, Ana Berbel, Carlos Romero, Elena Zuriaga

**Affiliations:** ^1^ Centro de Citricultura y Producción Vegetal, Instituto Valenciano de Investigaciones Agrarias (IVIA), Moncada, Valencia, Spain; ^2^ Instituto de Biología Molecular y Celular de Plantas (IBMCP), Consejo Superior de Investigaciones Científicas (CSIC)-Universidad Politécnica de Valencia (UPV), Valencia, Spain

**Keywords:** PPV, apricot, silencing, MATHd, *NbPMC*, orthologs, Rosaceae, *Prunus*

## Abstract

Sharka disease, caused by the *Potyvirus plumpoxi* (plum pox virus, PPV), is the primary limiting factor for stone fruit production globally, and the development of PPV-resistant cultivars is the most effective long-term strategy for controlling this disease. Recent studies have identified the *Prunus armeniaca PPVres* MATHd-containing (*ParPMC*) genes, part of a cluster of similar genes, as key host susceptibility factors essential for PPV infection in apricot. However, their specific functions remain largely unknown. This study examined the spatial expression patterns of the *ParPMC1* and *ParPMC2* genes, showing that they were primarily expressed in vascular bundle-rich tissues and were downregulated in resistant apricot cultivars. At subcellular level, both proteins localized in the nucleus and cytoplasm but ParPMC1 was distributed throughout the nucleus, whereas ParPMC2 appeared to be confined to the nuclear envelope. Orthology analyses revealed a “one-to-many” topology, indicating that a single ancestral gene duplicated after the emergence of the Rosaceae family, followed by additional tandem duplications and losses within *Prunus* species. To assess whether *ParPMC* downregulation contributed to PPV resistance, the *ParPMC* ortholog in *Nicotiana benthamiana* (*NbPMC*) was efficiently silenced using Tobacco Rattle Virus (TRV)-mediated Virus-Induced Gene Silencing (VIGS), resulting in a reduction in PPV infection. Overall, these results support the initial hypothesis that *ParPMC1* and/or *ParPMC2* function as host susceptibility genes in apricot, and their silencing may confer resistance to PPV. Moreover, their expression in conductive tissues suggests a potential role in the long-distance movement of the virus. This study marks an important first step in characterizing *ParPMC* genes and their role in PPV infection.

## 1 Introduction

Sharka, caused by the *Potyvirus plumpoxi* (plum pox virus, PPV), is the most devastating viral disease for *Prunus* species worldwide (García et al., 2014), with estimated costs since its emergence in Bulgaria in 1917–1918 likely exceeding €13x10^9^ (Cambra et al., 2024). PPV is recognized as one of the ‘Top 10 Viruses’ due to its scientific and economic importance (Scholthof et al., 2011) and is included in the EPPO A2 list of pests recommended for regulation as quarantine pests (EPPO, 2024). Recently, accumulated knowledge about PPV, including its hosts, control methods, and its applications in biotechnology has been reviewed (García et al., 2025). PPV belongs to the *Potyvirus* genus within the *Potyviridae* family, the largest genus of plant viruses, which causes significant losses in a wide variety of crops (Revers and García, 2015). *Prunus* species cultivated for fruit production (stone fruit trees) are the primary woody hosts, although the virus also affects wild and ornamental *Prunus* species and some herbaceous plants (García et al., 2014, 2025). To date, phylogenetic analyses have identified 10 independent strains, each exhibiting a preference for specific hosts (García et al., 2025). Among them, the Dideron (D) strain is the most prevalent, primarily infecting apricots and plums, while the Marcus (M) strain triggers rapid epidemics in peaches, suggesting a degree of host adaptation (Šubr and Glasa, 2013). Nonetheless, both strains can infect all three species (García et al., 2025).

Development of PPV-resistant cultivars appears to be the most efficient and sustainable long-term solution to combat the Sharka disease. Extensive germplasm screenings have identified only a few sources of resistance: a limited number of apricot (*Prunus armeniaca* L.) cultivars from North America and Azerbaijan (Martínez-Gómez et al., 2000; Polo-Oltra et al., 2020; Rakida et al., 2023), some wild apricots from Central Asia (Decroocq et al., 2016), the almond (*Prunus dulcis* L.) cultivar ‘Garrigues’ (Rubio et al., 2003), some tolerant European plums (*Prunus domestica* L.) exhibiting a hypersensitive response (HR) to PPV infection (Neumüller et al., 2005), and the peach (*Prunus persica* (L.) Batsch)-related *Prunus davidiana* clone P1908 (Pascal et al., 1998). Nonetheless, the specific mechanisms underlying resistance in each case remain poorly understood. For example, the transfer of PPV resistance from the almond cultivar ‘Garrigues’ to the peach rootstock ‘GF305’ through grafting (Rubio et al., 2003) has recently been linked to a robust RNA silencing antiviral response (Rodamilans et al., 2022). On the other hand, despite ongoing research, the host factors involved in HR-mediated resistance in the European plum cv. ‘Jojo’ remain unidentified (Rodamilans et al., 2014). Moreover, a new PPV isolate has been discovered that bypasses this resistance. This isolate contains alterations in three amino acids within the NIa region of the viral polyprotein, which are crucial for facilitating the HR-escaping response (Rodamilans et al., 2023). In the case of the *P. davidiana* clone P1908, linkage and association mapping suggest a complex pattern of polygenic inheritance, but the genetic factors involved are still unknown (Cirilli et al., 2017; Decroocq et al., 2005; Marandel et al., 2009; Rubio et al., 2010). Regarding apricot, years of dedicated efforts by different research groups have supported the presence of the major dominant *PPVres* locus in the upper part of apricot linkage group 1 (Dondini et al., 2011; Lalli et al., 2008; Lambert et al., 2007; Marandel et al., 2009; Mariette et al., 2016; Pilarova et al., 2010; Soriano et al., 2008; Vera-Ruiz et al., 2011). This locus comprises a cluster of genes containing meprin and TRAF-C homology domains (MATHd), which have been implicated in conferring resistance to PPV (De Mori et al., 2019; Rubio et al., 2015; Zuriaga et al., 2013, 2018). Within this cluster, subsequent genomic and transcriptomic analyses revealed *ParPMC1* and *ParPMC2* as host susceptibility genes required for PPV infection in apricot (Zuriaga et al., 2013, 2018). Both genes, named as *Prunus armeniaca PPVres* MATHd-containing genes (*ParPMC*), appeared clearly down-regulated in resistant cultivars, and showed genomic variants linked in coupling to PPV resistance. However, their function and underlying mechanism of action remain unclear.

The MATH domain, also known as TRAF (TNF-Receptor Associated Factor), is a fold of seven antiparallel β-helices that participates in protein-protein interactions (Zapata et al., 2007). Based on their association with other protein-domains, MATHd-containing proteins have been classified into different families or classes (Qi et al., 2022; Zapata et al., 2007). Among them, MATHd-only proteins, which contain one to four MATH domains without any other associated domains, form the largest cluster of MATHd-containing proteins in *Arabidopsis thaliana* (Qi et al., 2022). The apricot ParPMC1 and ParPMC2 proteins belong to this MATHd-only family, each possessing two MATH domains. The MATHd-only proteins are known to function as adaptors involved in signal transduction for diverse physiological processes (Qi et al., 2022; Zapata et al., 2007), but the function of many of these proteins remains unknown. Among them, *MUSE13* and *MUSE14* (*mutant, snc1-enhancing*) are susceptibility genes that encode factors required for pathogen infection (Huang et al., 2016). These redundant *A. thaliana* proteins have been identified as molecular adaptors associated with E3 ligases, which regulate immune response and autophagy via ubiquitination and degradation of different downstream substrates (Huang et al., 2016; Qi et al., 2017, 2022). Remarkably, loss of their function leads to enhanced pathogen resistance and autoimmunity (Huang et al., 2016). On the other hand, the *RTM3* gene, which encodes a distinct type of MATH protein (belonging to the MATH-PEARLI-4 class), has been identified as playing a role in restricting the long-distance movement of PPV and other potyviruses in *A. thaliana* (Cosson et al., 2010). *RTM3* is one of the three dominant *Restricted Tobacco-etch virus Movement* (*RTM*) genes identified in *A. thaliana*, along with *RTM1* (a jacalin) and *RTM2* (a small heat shock-like protein). Contrary to *ParPMC1* and *ParPMC2*, the non-functionality of one or more *RTM* alleles is sufficient to abolish the resistance phenotype (Cosson et al., 2010). Both *ParPMC* and *RTM3* belong to a MATHd-containing gene cluster in their respective genomic regions, though previous analyses showed these regions are not syntenic (Zuriaga et al., 2018).

To further investigate the function of *ParPMC* genes and their role in PPV resistance in apricot, we used complementary approaches. We analyzed their tissue-specific expression patterns and examined the conservation of their associated tandem gene cluster across different species through phylogenetic analysis. Additionally, we used *Nicotiana benthamiana* as a heterologous host model to study the potential role of the *ParPMC* ortholog in the PPV resistance using virus-induced gene silencing (VIGS). Our findings enhance understanding of the apricot-PPV pathosystem and offer valuable insights for developing new strategies to improve PPV resistance.

## 2 Materials and methods

### 2.1 RNA extraction and RT-qPCR analysis of apricot genes expression

Ten apricot cultivars were used for RT-qPCR analyses: 5 PPV resistant (‘Goldrich’, ‘Harlayne’, ‘Lito’, ‘Veecot’ and ‘Harcot’) and 5 PPV susceptible (‘Canino’, ‘Ginesta’, ‘Katy’, ‘Mitger’ and ‘Tadeo) (Martínez-Gómez et al., 2000). All of them are adult trees maintained as part of the germplasm collection at IVIA (Moncada, Valencia, Spain). Tissues analyzed (leaf blade, petiole, and primary and secondary veins) were randomly collected from throughout the canopy of a single tree per accession in the IVIA orchard and immediately frozen in liquid nitrogen between May and June 2019.

Total RNA was isolated from 60 mg of powdered tissues with the Plant/Fungi Total RNA Purification Kit (Norgen Biotek Corp., Thorold, Canada), adding polyvinylpyrrolidone (2% w/v PVP-40) and β-mercaptoethanol (2% v/v) to the kit extraction buffer, and then treated with the RNase-Free DNase I Kit (Norgen Biotek Corp., Thorold, Canada). RNA quality and quantity were checked by agarose gel electrophoresis, Nanodrop ND-1000 spectrophotometer (Nanodrop Technologies, Wilminto, DE, USA) and Qubit^(R)^ RNA BR Assay Kit (Thermo Fisher Scientific, Waltham, MA, USA).

Total RNA (500 ng) was reverse transcribed with the PrimeScript RT reagent kit using an Oligo-d(T) primer (Takara Bio, Otsu, Japan) in a total volume of 10 μl. RT-qPCR was performed on a StepOnePlus Real-Time PCR System (Life Technologies, Carlsbad, CA, USA), using SYBR premix Ex Taq (Tli RNaseH plus) (Takara Bio Inc, Shiga, Japan). Primer pairs are listed in **Supplementary Table 1**. Cycling conditions included 10 min at 95°C, followed by 40 cycles of 15 s at 95°C and 1 min at 60°C. PCR specificity was confirmed by a single peak in the dissociation curve. Gene expression was normalized using the relative standard curve method and the geometric mean of *Actin* and *Sand*-like (Lloret et al., 2017), suitable for large sample sets and cross-tissue comparisons. Results were the average of 3 technical replicates each one. Multiple sample comparisons were assessed using the non-parametric Kruskal-Wallis test (95% confidence) in Statgraphics Centurion XVII v. 17.2.00 (Statpoint Technologies, Warrenton, VA, USA). Significantly different samples were labeled with different letters.

### 2.2 *In situ* hybridization

RNA *in situ* hybridization with digoxigenin-labeled probes was performed on 8 μm paraffin sections of petiole and leaf tissues of the PPV-susceptible apricot cultivars ‘Katy’ and ‘Mitger’ as described by Ferrándiz et al. (2000). Probes were PCR-amplified, using ‘Ginesta’ cDNA as template and the primers listed in **Supplementary Table 1** (336 bp of *ParPMC1* and 370 bp of *ParPMC2*, both fragments from the 3’ cDNA end), and cloned into the pGEM-T-Easy vector (Promega). Sense and antisense riboprobes were labeled with digoxigenin using the DIG RNA Labelling Kit (SP6/T7; Roche, Gipf-Oberfrick, Switzerland). Images were obtained using a Nikon microscope (Eclipse 80i).

### 2.3 Subcellular localization assay in *Nicotiana benthamiana*

The localization of ParPMC1 and ParPMC2 proteins was first determined in healthy tissue using a nuclear subcellular marker. The full coding regions of *ParPMC1* and *ParPMC2* were amplified from cDNA of ‘Katy’ petiole tissue using Phusion^®^ High-Fidelity DNA Polymerase (ThermoFisher) and specific primers with *NcoI/NheI* restriction sites (**Supplementary Table 1**). The genes were then cloned into the pSK35S-GFP:eGFP-PoPit vector (Leastro et al., 2015) through In-Fusion Cloning (Takara), replacing the GFP gene. Finally, the corresponding expression cassettes were subcloned into the pMOG_800_ binary vector (Knoester et al., 1998) using *SacI* restriction sites.

To investigate protein localization within cell nuclei, two leaves of 2-weeks old *N. benthamiana* plants were agroinfiltrated with *Agrobacterium tumefaciens* strain C58C1 cultures carrying the ParPMC1:eGFP or ParPMC2:eGFP (OD_600_ = 0.5) and the nucleus marker (SV40 large T antigen fused to the RFP) mixed in equal ratio (OD_600_ = 0.5). Subsequently, it was assessed whether this localization was altered during PPV infection. For this purpose, *N. benthamiana* plants were co-agroinfiltrated with *A. tumefaciens* carrying the pLX-PPVr binary plasmid (Rodamilans et al., 2021) mixed in equal ratio with the corresponding pMOG_800_ plasmid described earlier. All plants were maintained under controlled conditions of 23°C with 16 hours of light and 18°C with 8 hours of darkness, at 70% relative humidity. Fluorescence was monitored 2 days post-infiltration in a confocal laser scanning microscope LSM780 ZEISS (CARL ZEISS MICROSCOPY, Jena, Germany). GFP fusion proteins and chlorophylls were excited at 488 nm, with emission captured at 495–520 nm and 660–720 nm, respectively. RFP was excited at 552 nm, and emission was captured at 585–610 nm. Image processing and analysis, including overlays and Z-stack projections, were conducted using Fiji ImageJ software (v. 1.52). All displayed images are representative of at least three independent experiments.

### 2.4 Identification of putative orthologs of *ParPMC* genes in other species

The presence of putative orthologs of the *ParPMC* genes cluster was analyzed across 31 species (**Supplementary Table 2**) using a three-step approach. First, a Reciprocal Best Hit (RBH) analysis was carried out using standalone BLASTP 2.12.0+ through custom-made python scripts. According to the peach genome (v2.0), the cluster contains 9 MATHd-containing genes in this species. The predicted peach proteins were used as initial queries against the other protein databases and, subsequently, the 3 first hits obtained were reciprocally BLASTed against the peach proteins database. Second, the neighborhood of the identified RBHs was analyzed to detect syntenic blocks encompassing the *ParPMC* gene cluster across different genomes. The translated amino acid sequence of 3 genes upstream and downstream of each RBH found were used as queries to identify new RBHs pairs and to define synteny blocks. Third, phylogenetic tree-based analysis was conducted by MEGA11 software (Tamura et al., 2021). Amino acid sequences of the putative orthologous identified were aligned by MUSCLE (Thompson et al., 1994) and manually edited with Bioedit (Hall, 1999). Poorly aligned positions and divergent regions of the alignment were eliminated using Gblocks v.0.91b (Castresana, 2000). The best-fitting evolutionary model (JTT+G), according to the Akaike information criterion (AIC), was implemented in the Maximum Likelihood (ML) phylogenetic analysis (Felsenstein, 1985; Jones et al., 1992) using 100 bootstrap replications. Evolutionary divergences between sequences were estimated using MEGA11, with the same evolutionary model and all amino acid positions, but removing ambiguous positions for each sequence pair. To enhance clarity in this work—particularly in the phylogenetic tree—gene names have been simplified. The putative orthologs identified have been coded using a binomial nomenclature that begins with the initials of the species followed by a letter (A to I) indicating their positions within the cluster. In cases where multiple cultivars of the same species are present, their names have also been indicated. Expanding this system to accommodate a larger number of cultivars, nevertheless, would require the development of an alternative nomenclature, which falls outside the scope of this study.

### 2.5 Silencing the *NbPMC* gene using VIGS and its effects on PPV infection

For VIGS, pTRV1 and pTRV2 gateway vectors (Liu et al., 2002) were used. A 300 nt fragment of the *NbPMC* gene (4th exon) was selected using the SGN VIGS tool (Fernandez-Pozo et al., 2015). Fragment was PCR amplified using gene-specific primers (**Supplementary Table 1**). Besides, a 300 bp fragment of *mGFP5* gene was amplified as described by Navarro et al. (2020) and assembled with the *NbPCM* fragment by overlap extension PCR. Both fragments, *NbPMC* and *GFP:NbPMC*, were cloned into the pDONR207. The resultant pENTRY vectors were recombined with pTRV2 to get the VIGS clones pTRV2[NbPMC] and pTRV2[GFP: NbPMC] according to the supplier’s instructions (Invitrogen Life Tech, Carsland, CA, USA). Moreover, pTRV2[GFP], a pTRV2 containing the whole *mGFP5* gene, was used as a control (Navarro et al., 2020). pTRV1 and all pTRV2 derivatives were transferred into *A. tumefaciens* strain C58C1 by electroporation. Transformed bacteria were grown overnight at 28°C shaking at 200 rpm in Luria-Bertani (LB) medium with kanamycin and rifampicin antibiotics. *A. tumefaciens* cells were harvested and resuspended in infiltration media (10 mM MgCl2, 10 mM MES, and 150 μM acetosyringone), and adjusted to an OD_600_ of 1.

Two-week-old *N. benthamiana* wild-type and GFP16c line (Ratcliff et al., 2001) plants, grown under long-day photoperiods (16 h light at 25°C and 8 h dark at 22°C), were used for VIGS experiments. Two leaves per plant were infiltrated into the abaxial side, using 1:1 (v/v) mixtures of two *Agrobacterium* cultures harboring the pTRV1 and one of the three pTRV2-derived vectors. Ten days later, two non-infiltrated upper leaves per plant were mechanically inoculated by finger-rubbing with 20 μl of extracts prepared by grinding 500 mg of leaves from *N. benthamiana* plants infected with RFP-tagged PPV (pLX-PPVr; Rodamilans et al., 2021) in 2 ml of 30 mM phosphate buffer (pH 7.0) supplemented with carborundum. Three plants per construct were used.

Gene silencing and PPV viral RNA expression levels were monitored by RT-qPCR (primers listed in Additional File 1) at six days post-inoculation, as previously described in the first section. PPV-inoculated (local) and upper PPV-non-inoculated (systemic) leaves were collected, ground in liquid nitrogen and stored at -80°C until subsequent analyses. The results represent the average of 3 independent biological replicates, each with 3 technical replicates. Relative expression levels were calculated using the comparative Ct (ΔΔCt) method described by Livak and Schmittgen (2001). Protein phosphatase 2A (PP2A, TC21930) and F-box (Niben.v0.3.Ctg24993647) genes were used as endogenous controls to normalize the expression levels (Liu et al., 2012). Primer efficiencies were tested using serial dilutions of the corresponding cDNA. Significant differences were determined using Student’s t-test (p<0.05).

## 3 Results

### 3.1 *ParPMC* genes are preferentially expressed in vascular-enriched tissues in apricot and down-regulated in resistant cultivars

**Figure 1:**
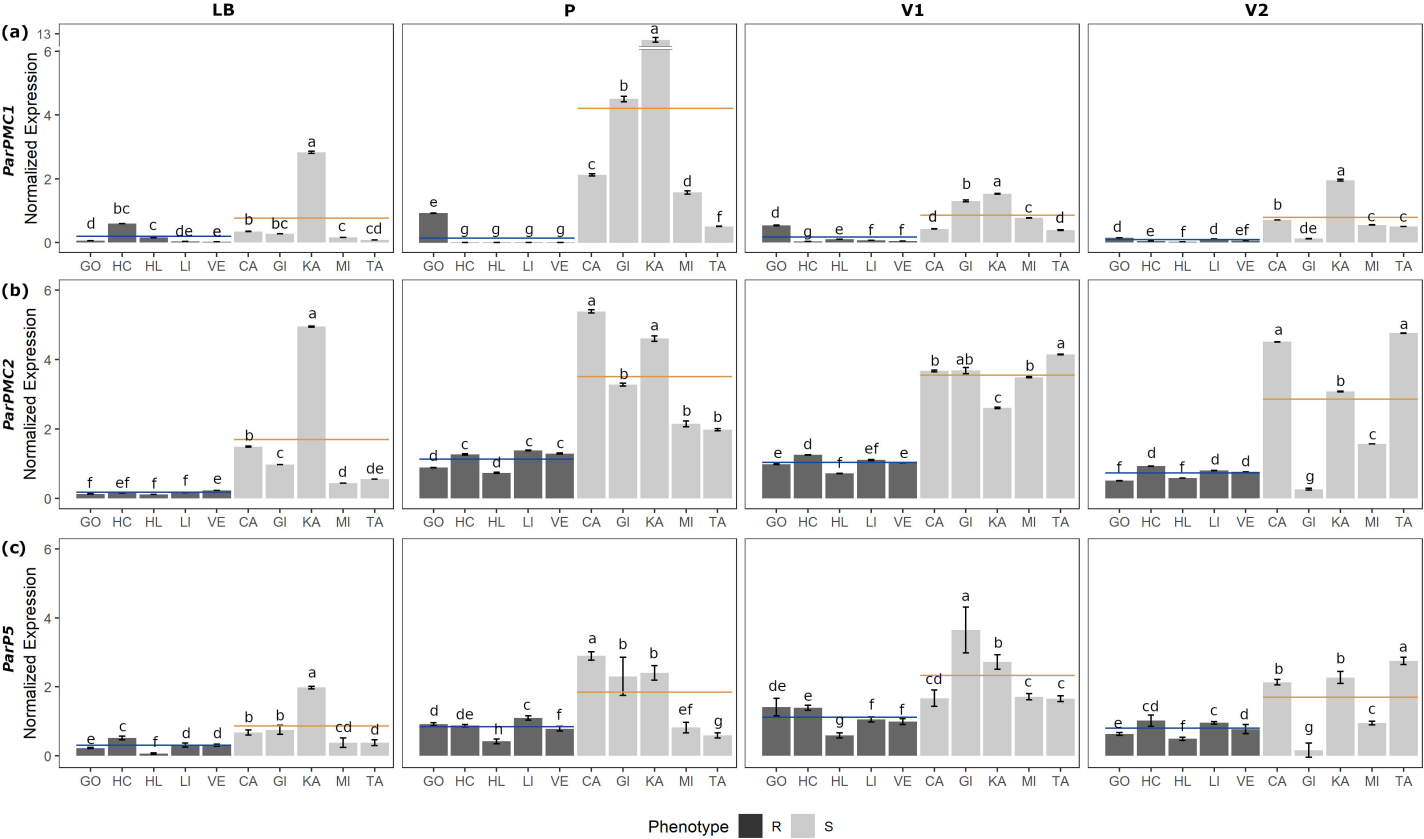
RT-qPCR analysis of *ParPMC1* **(a)**, *ParPMC2* **(b)** and *ParP5* **(c)** genes in leaf blade (LB), petiole (P), primary (V1) and secondary veins (V2), respectively. Error bars represent standard deviation, and different letters denote significant differences (P<0.05). Resistant (R; GO: ‘Goldrich’, HC: ‘Harcot’, HL: ‘Harlayne’, LI: ‘Lito’ and VE: ‘Veecot’) and susceptible (S; CA: ‘Canino’, GI: ‘Ginesta’, KA: ‘Katy’, MI: ‘Mitger’ and TA: ‘Tadeo’) apricot cultivars are indicated in black and gray, respectively. Blue and orange lines indicate R and S mean values, respectively.

Tissue-specific gene expression patterns of *ParPMC1*, *ParPMC2* and the adjacent gene *ParP5* were analyzed to gain insight into their specific functions and to provide context for their potential roles. *ParP5* was included as a control, as an adjacent paralog of both genes, with no resistance-associated genomic variants and exhibiting similar expression levels in full leaves of both resistant and susceptible varieties grafted onto PPV-D-infected GF-305 rootstocks (Zuriaga et al., 2013, 2018). In the present study, using material collected directly from the orchard, we conducted detailed RT-qPCR analyses on leaf blades, petioles, primary and secondary veins from five resistant (‘Goldrich’, ‘Harcot’, ‘Harlayne’, ‘Lito’, and ‘Veecot’) and five susceptible apricot cultivars (‘Canino’, ‘Ginesta’, ‘Katy’, ‘Mitger’, and ‘Tadeo’) (**Figure 1**; **Supplementary Table 3**).

Although variability among the accessions was observed, on average, *ParPMC1* and *ParPMC2* showed statistically significantly higher expression in susceptible cultivars than in resistant ones across all tissues (**Figures 1a, b**, respectively; **Supplementary Table 3**), except for *ParPMC1* in the leaf blade. In contrast, *ParP5* did not show consistent differences between susceptible and resistant cultivars (**Figure 1c**; **Supplementary Table 3**). *ParPMC1* exhibited the highest expression levels in petiole samples, with a marked difference between susceptible and resistant plants—expression was on average 22.4 times higher in the susceptible ones. In the primary and secondary veins, *ParPMC1* expression was approximately 5 and 10 times higher in the susceptible plants compared to the resistant ones, respectively. It is worth noting that *ParPMC1* expression in resistant varieties was the lowest of the three genes analyzed. Regarding *ParPMC2*, similar expression patterns were observed in the petiole and both primary and secondary veins, with 3–4 times higher expression in susceptible cultivars compared to resistant ones, while the expression was generally lower in the leaf blade.

**Figure 2:**
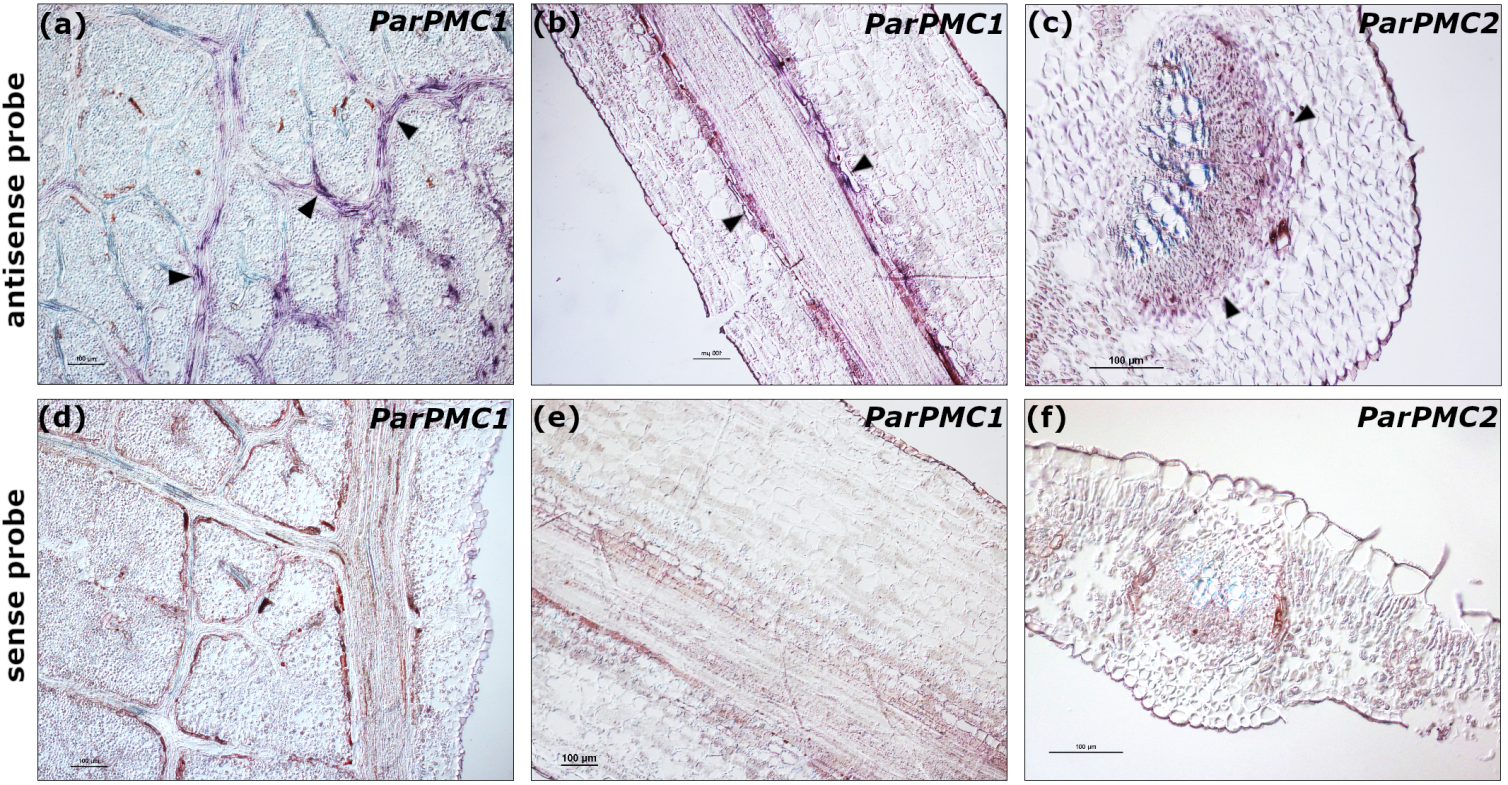
*In situ* hybridization to detect *ParPMC1* (**a**: longitudinal leaf section, **b**: longitudinal petiole section) and *ParPMC2* (**c**: transverse leaf section) transcripts using antisense DIG-labeled RNA probes. Arrows indicate a positive blue-violet signal localized in the veins and vascular bundles. Negative controls using sense probes are indicated **(d-f)**. Bars: 100µm. The images are representative of the range of variability observed.

To analyze the spatial expression pattern of the *ParPMC* genes, *in situ* hybridization was performed on leaf and petiole sections from the PPV-susceptible apricot cultivars ‘Katy’ and ‘Mitger’. Microscopic examination revealed that both *ParPMC1* and *ParPMC2* transcripts were primarily localized in the veins and vascular bundles (**Figures 2a–c**). As expected, no signal was detected in samples hybridized with the sense probe (**Figures 2d–f**).

### 3.2 At the subcellular level, ParPMC1 and ParPMC2 proteins localize in the nucleus and the cytoplasm

**Figure 3:**
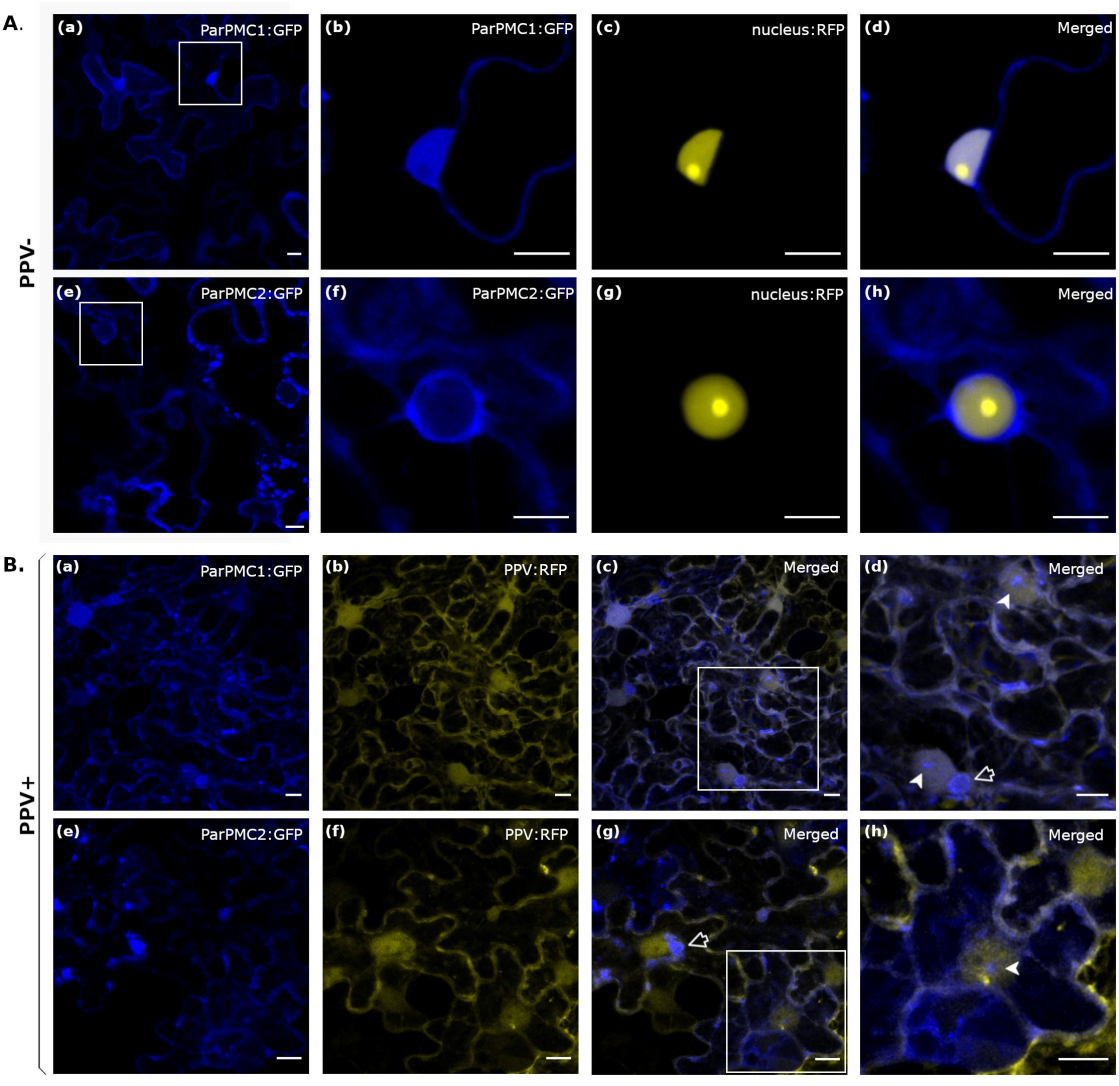
Subcellular localization of ParPMC1 and ParPMC2 proteins in *N. benthamiana* leaf epidermal cells in absence **(A)** and presence **(B)** of PPV infection. Fluorescence was captured at 48 hpi using a confocal microscope Zeiss LSM 780 model. **(A)**: ParPMC:GFP was co-expressed with the nuclear localization signal of SV40 large T antigen fused to the RFP. The blue (GFP) **(a, b, e, f)**, yellow (RFP) **(c, g)** and merged channels **(d, h)** are shown. **(B)**: ParPMC:GFP was co-infiltrated with PPV expressing the RFP reporter. The blue (GFP) **(a, e)**, yellow (RFP) **(b, f)**, and merged channels **(c, d, g, h)** are shown. Empty arrows indicate perinuclear aggregates, while filled arrows point to nuclear structures. All images correspond to Z-stack projections and are representative of the range of variability observed. Bars: 10µm.

Transient expression of both ParPMC1 and ParPMC2 proteins fused to the GFP protein in leaves of healthy *N. benthamiana* plants showed nuclear and cytoplasmic localization in both cases (**Figure 3A**). However, co-expression with a nuclear marker revealed slightly different behaviors for the two proteins. While ParPMC1 is distributed throughout the nucleus (**Figures 3Aa–d**), ParPMC2 appears confined to the nuclear envelope (**Figures 3Ae–h**). To evaluate changes in their distribution upon PPV infection, the analysis was repeated in *N. benthamiana* plants infected with RFP-labeled PPV (Rodamilans et al., 2021) (**Figure 3B**). The virus altered the localization of both ParPMC1 and ParPMC2, with ParPMC1 entering the nucleolus (**Figures 3Ba–d**), and ParPMC2, in certain cells, being detected inside the nucleus (**Figure 3Bh**). Moreover, both proteins appear to form perinuclear aggregates (**Figures 3Be–g**). Additionally, aggregates were observed near the plasma membrane, which could be nonspecific due to overexpression. Nevertheless, the exact localization requires confirmation through additional experiments.

### 3.3 A gene duplication and subsequent diversification in Rosaceae led to the formation of a tandemly arrayed *PMC* gene cluster in *Prunus* species

**Figure 4:**
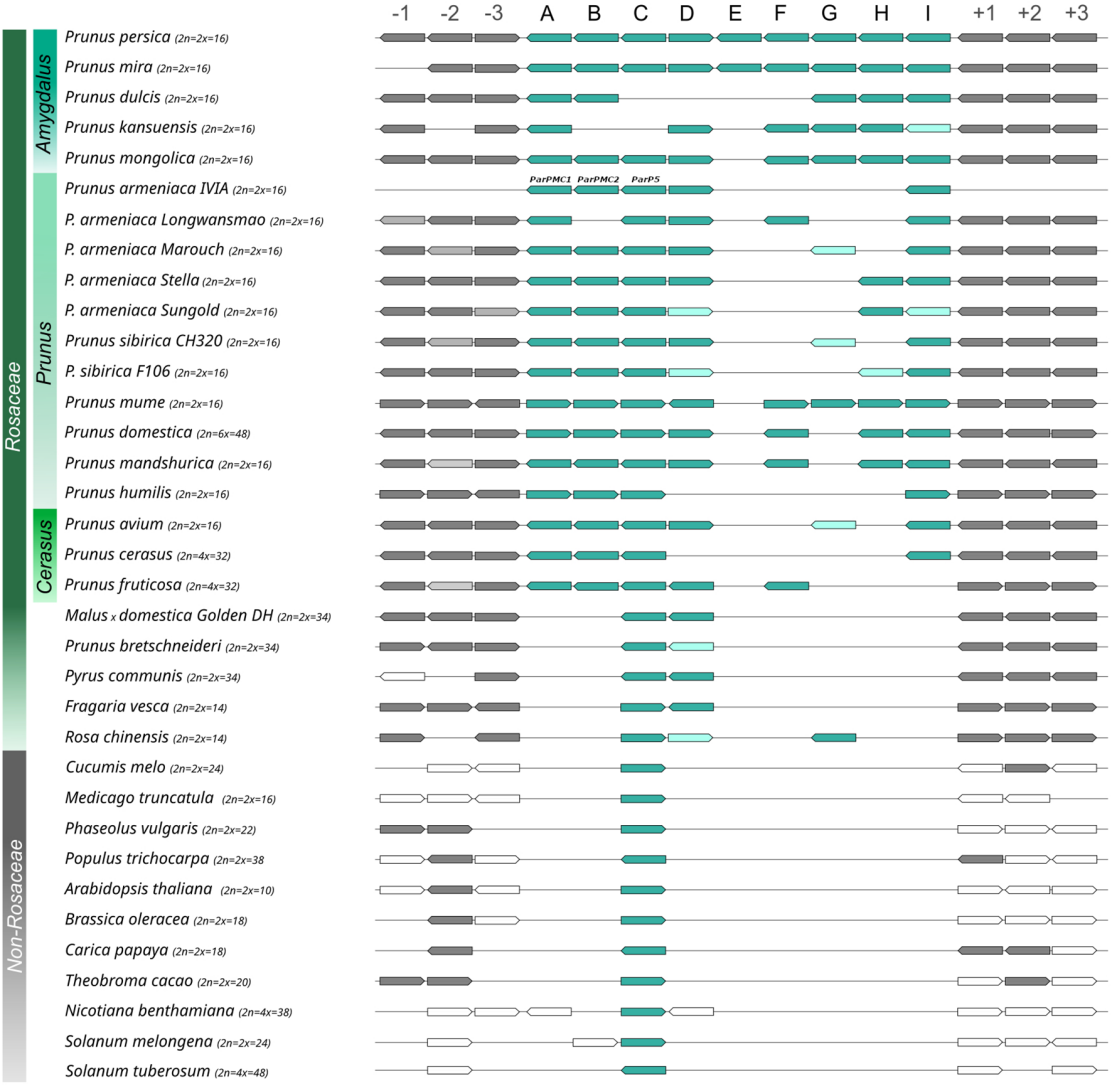
Schematic representation of the identified putative orthologs, with MATHd cluster genes in blue and flanking region genes in gray. Dark and light colors represent BRHs and second hits from the reciprocal BLASTp, respectively.

A three-pillar strategy was designed to identify putative orthologs of the tandemly arrayed MATHd genes, including the *ParPMC* genes, in 35 genomes of 31 species (**Supplementary Table 4**). Although focusing particularly on *Prunus* spp., the analysis included 19 species from the Rosaceae family (i.e. 14 *Prunus* spp., 1 *Malus*, 2 *Pyrus* spp., 1 *Fragaria*, and 1 *Rosa*), as well as representatives from other families (i.e. 2 Brassicaceae, 1 Caricaceae, 2 Cucurbitaceae, 2 Fabaceae, 1 Malvaceae, 1 Salicaceae and 3 Solanaceae). In addition, the assembled transcripts identified in our previous RNA-seq analysis (Zuriaga et al., 2018) were also included. First strategy relied on Reciprocal Best BlastP Hit (RBH) search comparing all-to-all protein databases (Zheng et al., 2005). Since the peach genome is considered as the reference for *Prunus* genome analysis, the 9 proteins encoded by the peach cluster of MATHd-containing genes were used as queries against the other protein databases. The top 3 hits were then reciprocally BLASTed against the peach protein database, resulting in the identification of RBHs (**Supplementary Table 2**). The second strategy explored the conservation of the genomic region containing the MATHd genes cluster in other species by analyzing the three closest flanking genes, both upstream and downstream, as suggested by Zheng et al. (2005). The third strategy aimed to infer the phylogenetic relationships between the obtained sequences. As previously mentioned, to enhance clarity in this work—particularly in the phylogenetic tree—the gene names of the putative orthologs have been simplified using a binomial nomenclature: the initials of the species followed by a letter (A to I) indicating their position within the cluster (**Figure 4**; **Supplementary Table 2**).

The analysis revealed several misannotations, some of which have been corrected through re-annotation using BlastP (**Supplementary Table 2**). In most cases, a gene was misannotated because it actually comprised two adjacent genes. When possible, we reannotated them based on the BlastP results against peach, separating the two genes by adding ‘_a’ or ‘_b’ to their original names to indicate whether they correspond to the beginning or the end of the originally annotated protein, respectively. For instance, the gene *PruarM.1G134300*, originally annotated as encoding a 603-amino-acid protein in the *P. armenica* cv. ‘Marouch’ genome, was reannotated as *PruarM.1G134300_a* (*PruarC_Ma*) and *PruarM.1G134300_b* (*PruarB_Ma*), as these correspond to orthologs of *PrupeC* and *PrupeB*, respectively. Some other genes appear to be incomplete, as in the case of *PaJTYG0100001312.01* (*PruarI_Su*) in *P. armeniaca* cv. ‘Sungold’ or *PaF106G0100001271.01* (*PrusiD_F106*) in *Prunus sibirica* F106. Final sequences used for the phylogenetic analysis are included as **Supplementary Information** (**Supplementary File 1**).

Overall, RBH analysis revealed the presence of at least one ortholog of a MATHd gene in all analyzed species, except *Cucurbita pepo* (**Supplementary Table 2**). Interestingly, the MATHd gene cluster was found exclusively within the Rosaceae family, with a variable number of genes, while only a single orthologous gene (the *C* gene) was present in the other families (**Figure 4**). In *Prunus* spp., the number of genes in the MATHd gene cluster ranged from 4 to 9 genes, while in other analyzed *Rosaceae* species it only contained genes *C* and *D*, except for *Rosa chinensis*, which also had *RoschG*. Notably, the *E* gene was only present in peach (*PrupeE*), while a truncated fragment appeared in *P. mira* (*PrumiE*, reannotated as *Pmi01g1113_b*). However, since non-conserved domains were identified in *PrupeE* and it might not be properly annotated, this gene was excluded from the rest of the analysis.

**Figure 5:**
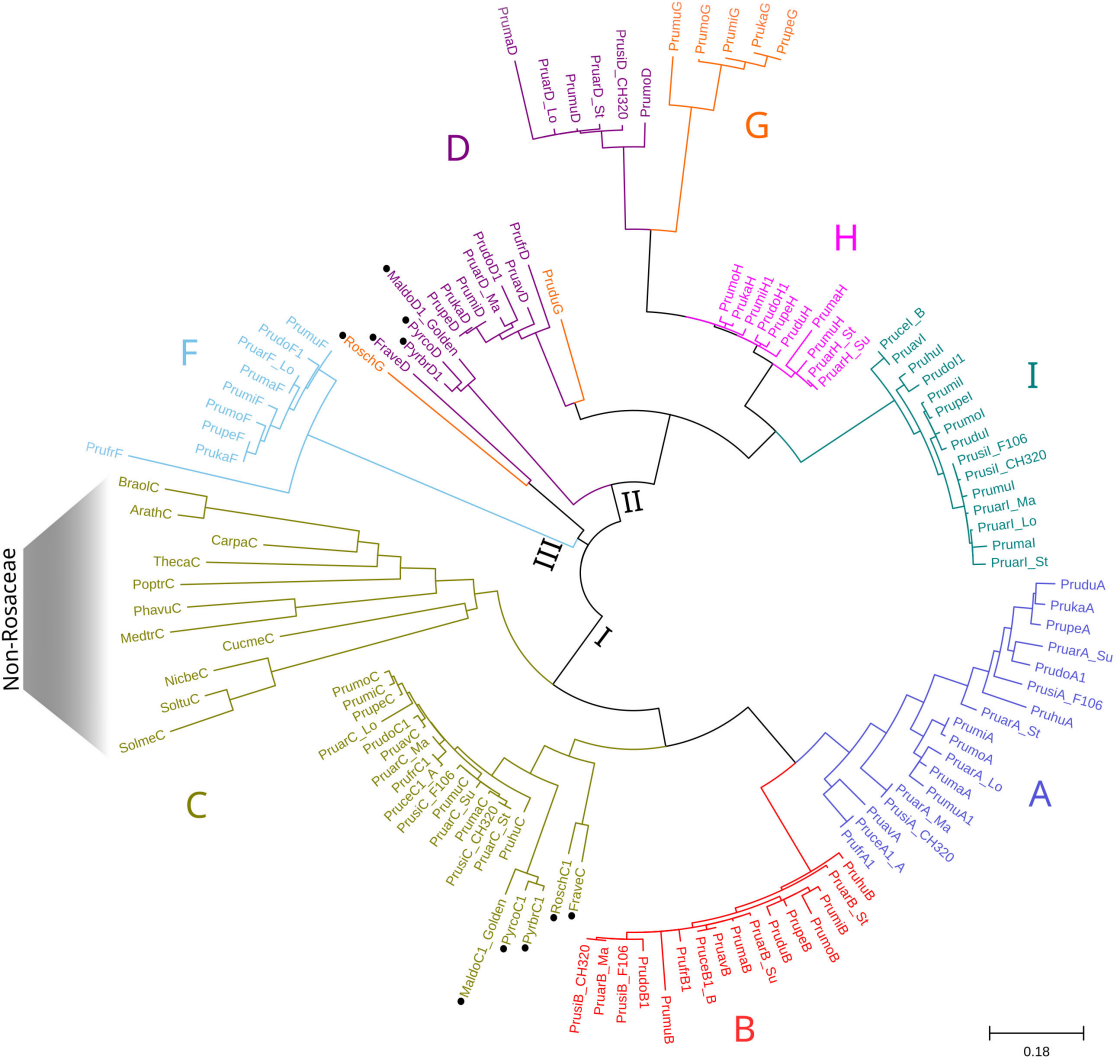
Maximum Likelihood phylogenetic tree of the MATHd gene cluster orthologs. The Jones-Taylor-Thornton (JTT) + G model was used as the best-fitting evolutionary model (287 aa), with branch lengths representing substitutions per site. Black circles indicate non-Prunus species. Letters A–I correspond to the position of the proteins within the cluster, consistent with **Figure 4**. The three major clusters identified are indicated at internal nodes using Roman numerals.

The maximum likelihood-based phylogeny of the 124 proteins identified as RBHs revealed three major clusters, which were further grouped according to the A-I classification of orthologous proteins (**Figure 5**). For clarity purposes, bootstrapping support values can be observed in more detail in the phylogenetic tree enclosed as **Supplementary File 2**. The first cluster includes the A, B, and C proteins, the second cluster contains the D, G, H, and I proteins, and the third cluster consists solely of F proteins. Within the first cluster, the C proteins from all non-Rosaceae species are positioned basally, separated from the rest of the C proteins. Following this, there is a subcluster of the remaining C proteins (with non-*Prunus* sequences separated), along with another subcluster of the A and B proteins. In the second cluster, the RoschG and FraveD proteins occupy a basal position, while the remaining D and G proteins are more dispersed. The H and I proteins each form monophyletic groups. Lastly, the third cluster is composed of the F proteins, which are not present in all *Prunus* species, and PrufrF appears more distantly related than the others.

The average evolutionary divergence within each protein group was estimated (**Supplementary Table 5**), with variability being significantly higher when sequences from non-*Prunus* species were included. The overall average evolutionary divergence across all sequence pairs was calculated to be 0.52. Among *Prunus* species, the A proteins exhibited the greatest variability (0.14), followed by the D and F proteins with a diversity index of 0.09 each. In contrast, the B and C proteins showed much lower variability, with a diversity index of 0.04 for both.

Additionally, as *MUSE13* and *MUSE14* are MATHd-containing genes required for pathogen infection in *A. thaliana* (Huang et al., 2016), their synteny with peach was also analyzed. BLASTP analysis of MUSE13 and MUSE14 against the Peach Genome v2.0.a1 transcript peptides revealed similarities in both cases with Prupe.1G033200.1, a protein containing one MATH domain and another domain named DUF5585 of unknown function, located at position Pp01:2300788.2311356 in the peach genome. MUSE13 (encoded by the *AT1G04300.3* transcript) showed a 53.68% similarity over 1170 aa of the alignment length (E-value = 0), while MUSE14 (encoded by the *AT5G43560.1* transcript) showed a 51.98% similarity over 1160 aa (E-value = 0).

### 3.4 *NbPMC* silencing reduces susceptibility to PPV in *Nicotiana benthamiana*

**Figure 6:**
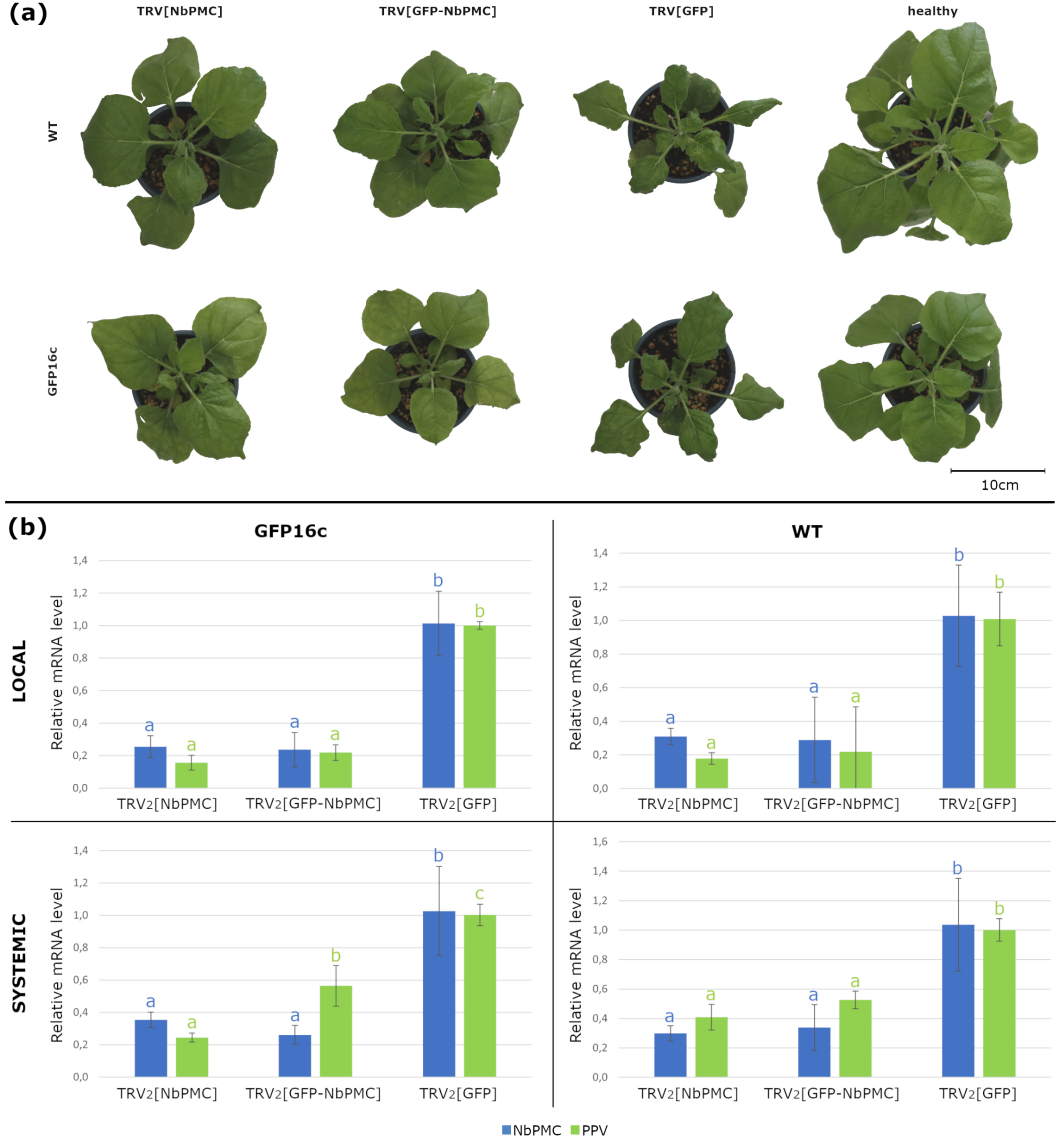
TRV-VIGS experiments to silence the *NbPMC* gene. **(a)** Wild-type (WT) and GFP16c *N. benthamiana* plants at 6 dpi with pTRV1 and pTRV2[NbPMC], pTRV2[GFP:NbPMC], pTRV2[GFP] and non-inoculated. Scale bar = 10 cm; **(b)** Relative expression levels of *NbPMC* (blue) and PPV (green) in local and systemic leaves of *NbPMC*- and *GFP:NbPMC*-silenced plants compared to *GFP*-silenced controls. Different letters indicate statistically significant differences (P < 0.05).

Due to the challenges of the *Prunus* transformation process, *N. benthamiana* has been used as a model organism to conduct functional analyses in this work. According to the synteny results, *Niben261Chr17g0988003.1 (NicbeC*) gene was the only ortholog of the *ParPMC* genes in this species. To maintain consistency with our previous study, *NicbeC* was renamed as *NbPMC* (*Nicotiana benthamiana PPVres* MATHd-containing genes), following the naming convention of the *ParPMC* genes (Zuriaga et al., 2018). Thereafter, to evaluate its potential role in PPV infection, *NbPMC* was downregulated using the tobacco rattle virus (TRV)-based VIGS method (Senthil-Kumar and Mysore, 2014). For this purpose, two different constructs, pTRV2[NbPMC] and pTRV2[GFP:NbPMC], were initially tested in GFP16c *N. benthamiana* plants (constitutively expressing GFP). To reduce off-target effects, the *NbPMC* silencing fragment was carefully designed to minimize sequence homology with other *N. benthamiana* transcripts. After confirming the efficacy of the constructs in this background, the experiment was subsequently repeated using wild-type (WT) plants. In each case, two leaves of three plants were agroinfiltrated with pTRV1 and either pTRV2[GFP], which was used as a control, pTRV2[NbPMC] or pTRV2[GFP:NbPMC]. Ten days later, PPV expressing the RFP reporter (pLX-PPVr; Rodamilans et al., 2021) was mechanically inoculated. At this stage, all three plant types—control (GFP-silenced), NbPMC-silenced, and GFP:NbPMC-silenced—exhibited only typical TRV-associated symptoms, with no other notable differences among them. At 6 days post-infection (dpi), NbPMC-silenced and GFP:NbPMC-silenced plants were slightly less developed than healthy plants, but they were not as affected as the control plants (**Figure 6A**; **Supplementary Figures 1**, **2**). Throughout the experiment, all plants were closely monitored, and no evident developmental abnormalities or delays specifically associated with NbPMC silencing were detected. *NbPMC* gene expression and PPV accumulation were analyzed by RT-qPCR in inoculated (local) and non-inoculated (systemic) upper leaves (**Figure 6B**; **Supplementary Table 6**). Both constructs caused a significant reduction in *NbPMC* expression, though not complete, which was associated with a marked decrease in viral accumulation. The differences were statistically significant in all cases. Analysis of leaves inoculated with pTRV2[NbPMC] showed a 3.3-fold reduction in *NbPMC* levels in WT plants, which resulted in a 5.6-fold decrease in PPV infection. Notably, a reduction of nearly 4-fold in *NbPMC* mRNA levels in GFP16c plants led to a 6.4-fold decrease in viral accumulation. Similar outcomes were observed with pTRV2[GFP:NbPMC], where *NbPMC* mRNA levels were reduced by 3.5 to 4.3-fold in WT and GFP16c plants, respectively, and PPV content decreased by 4.6-fold in both. Regarding systemic infection, a reduction in viral load was observed in non-inoculated leaves as well. With pTRV2[NbPMC], an average 3.5-fold reduction in *NbPMC* levels in WT plants resulted in a 2.4-fold decrease in PPV accumulation. In GFP16c plants, where gene silencing was slightly less effective (2.9-fold), the reduction in infection was more pronounced (4.1-fold). For pTRV2[GFP:NbPMC], *NbPMC* levels were reduced by 3.1 to 3.9-fold in WT and GFP16c plants, respectively, reducing the viral load to 1.9 and 1.8-fold of the control levels.

**Figure 7:**
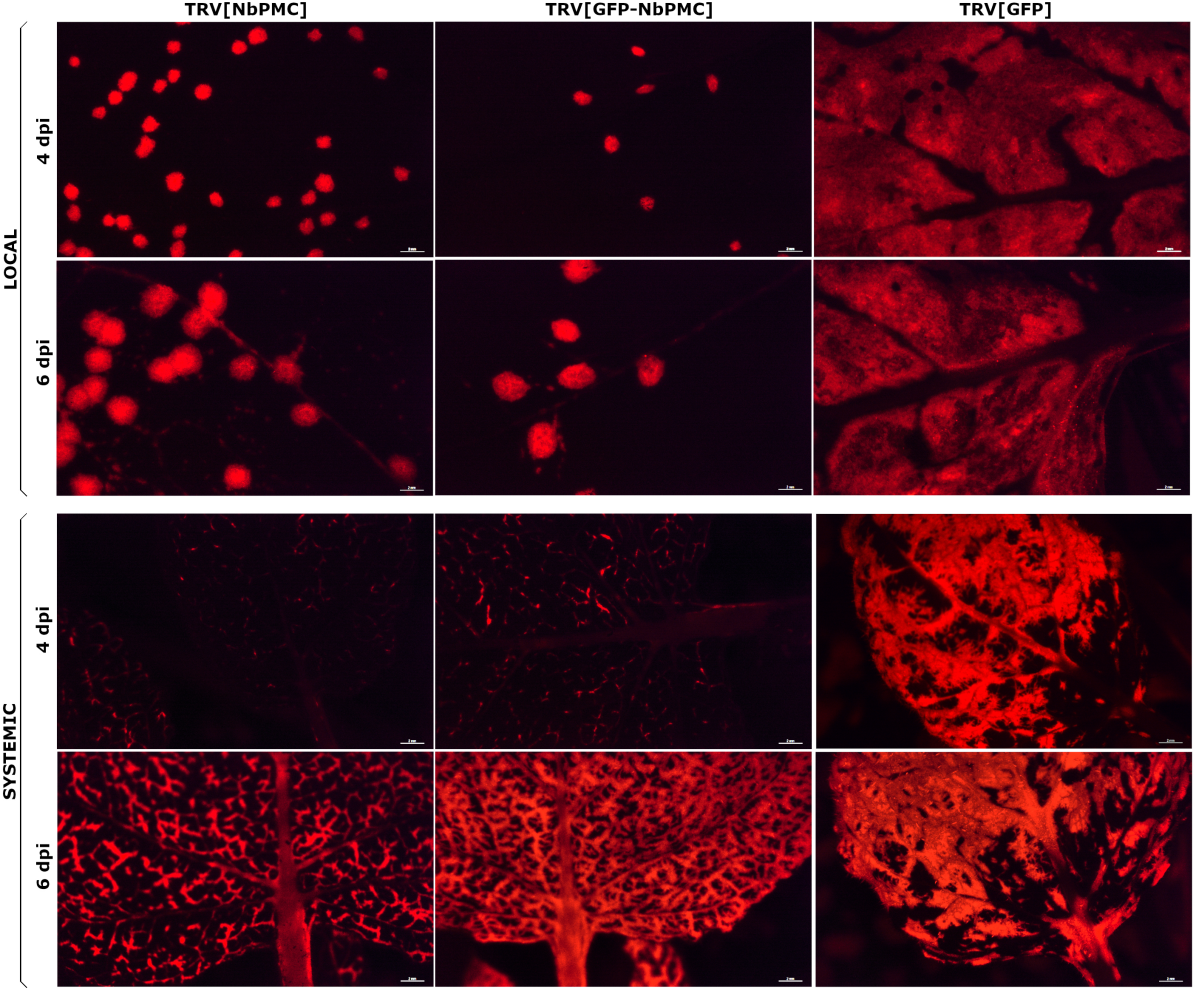
RFP-labeled PPV dispersion at 4 and 6 dpi in GFP16c *N. benthamiana* leaves of TRV-VIGS NbPMC-silenced, GFP:NbPMC-silenced, and control plants. RFP fluorescence was observed under a Leica MZ16F stereomicroscope. These images are representative of the range of variability observed.

Virus spread was analyzed in local and systemic leaves of NbPMC-silenced, GFP:NbPMC-silenced, and control plants (TRV[GFP]) at 4, 5 and 6 dpi in both WT and GFP16c genotypes (**Supplementary Figures 3**, **4**). A visual representation of the experiment using GFP16c plants for each construct at 4 and 6 dpi is shown in **Figure 7**. As observed, virus movement was faster in control plants (both WT and GFP16c), with PPV present throughout the leaf at 4 dpi in both inoculated and systemic areas. In contrast, in NbPMC-silenced plants, the virus remained confined to mesophyll cells of inoculated leaves at 4 dpi, with fewer foci in GFP:NbPMC-silenced plants. In systemic leaves, PPV was restricted to vascular tissues even at 6 dpi. This effect was more pronounced in the systemic leaves of NbPMC-silenced GFP16c plants, which also showed a lower viral RNA level compared to GFP:NbPMC plants (**Figure 7**).

## 4 Discussion

### 4.1 *ParPMC* are susceptibility genes to PPV preferentially expressed in transport tissues

*ParPMC* genes have been proposed as susceptibility factors for PPV infection in apricot, although their functions are still unclear (Zuriaga et al., 2018). In this study, we aimed to characterize these genes to enhance our understanding of their role in the infection process. For this purpose, we analyzed in detail their expression patterns and the subcellular location of their proteins. In agreement with previous experiments performed by Zuriaga et al. (2018), a significant down-regulation of *ParPMC1* and *ParPMC2* gene expression was again observed in PPV-resistant apricot cultivars compared to susceptible ones. A more detailed analysis of gene-expression across different tissues revealed that *ParPMC* genes were preferentially expressed in conductive tissues, particularly in veins and vascular bundles. These results support their potential involvement in the long-distance movement of PPV. Interestingly, previous studies have already shown that PPV movement is impaired in the resistant apricot cultivars (Dicenta et al., 2003; Ion-Nagy et al., 2006). Regarding the location of the virus, Dicenta et al. (2003) detected PPV particles in the xylem and sclerenchyma and Ion-Nagy et al. (2006) also found them in the phloem. Also, Collum et al. (2020) observed a strong response to PPV infection in the phloem tissues of European plum, which was linked to the activation of defense-related genes, including those involved in RNA silencing. Nevertheless, *ParPMC1* and *ParPMC2* are down-regulated in PPV-resistant cultivars regardless of PPV infection (Zuriaga et al., 2018) pointing out that they are not involved in an active plant response to the virus. The functions of most plant MATHd proteins are still largely unknown; however, some have been associated with biotic stresses (Ao et al., 2023; Huang et al., 2016; Qi et al., 2017, 2022). Among them, *MUSE13* and *MUSE14* are susceptibility genes, like the *ParPMC* genes in apricot, required for pathogen infection, with their loss enhancing resistance and autoimmunity (Huang et al., 2016). Notwithstanding, synteny analysis did not reveal an orthologous relationship between *MUSE13*/*MUSE14* and the *ParPMC* genes, resembling the lack of synteny observed with the dominant *RTM3* gene (Zuriaga et al., 2013).

The localization of ParPMC1 in the cell nucleus and ParPMC2 in the nuclear envelope, along with their modification in the presence of PPV, may provide insights into their potential roles in viral infection. By contrast, RTM3 was found in the cytosol and at the periphery of chloroplasts in *N. benthamiana* (Sofer et al., 2017), whereas MUSE13 and MUSE14 were detected in the cytosol and plasma membrane but were absent from the nucleus in *A. thaliana* (Huang et al., 2016). In this context, several conserved nuclear and nucleolar host factors required for virus infections in plants have been identified, such as importin α (Lukhovitskaya et al., 2015; Zhang et al., 2011), fibrillarin (Chang et al., 2016) and Exportin 1 (XPO1) (Zhang et al., 2021). Moreover, several potyviral proteins are known to localize not only in the cytoplasm but also in the nucleus, suggesting functional roles that depend on nucleocytoplasmic transport (Xue et al., 2023). For example, as described by Zhang et al. (2021), XPO1 is a key nuclear export receptor that facilitates the transport of the viral RNA-dependent RNA polymerase (NIb) of *Turnip mosaic virus* (TuMV) from the nucleus—where NIb is translocated after translation and undergoes SUMOylation to promote viral infection—toward the viral replication complexes (VRCs) located in the perinuclear region. This nucleocytoplasmic trafficking is essential for efficient viral replication. Notably, loss of XPO1 function in *Arabidopsis* and *N. benthamiana* significantly reduces TuMV replication and infection. Additionally, alterations in ParPMC1 and/or ParPMC2 localization in response to PPV may provide key insights into their involvement in viral infection. Notably, Cajal bodies (CBs)—subnuclear structures that may correspond to those observed sometimes for ParPMC2 in the presence of PPV—are known to be targeted during viral infection (Lettin et al., 2023). CBs play a crucial role in modulating certain viral infections, either by supporting viral replication or by enhancing the host’s defense mechanisms against viruses (Love et al., 2017). Ongoing experiments aim to refine the analysis of the subcellular localization of ParPMC1 and ParPMC2, as well as identify potential interactors, in order to gain a deeper understanding of the role of ParPMC proteins during PPV infection.

### 4.2 *ParPMC* genes are part of a specific *Prunus* tandemly arrayed gene cluster

Plant MATHd genes are frequently tandemly encoded, reminiscent of the genomic organization of the nucleotide-binding domain leucine-rich repeat-containing (NLR) receptors, a configuration believed to drive rapid diversification and adaptation under evolutionary pressure (Ao et al., 2023). Differences in the number of tandemly arrayed genes within the *ParPMC* cluster between apricot and peach were previously observed (Zuriaga et al., 2018). In this study, a detailed phylogenetic analysis of the cluster was performed, with a particular focus on *Prunus* species. The analysis revealed a ‘one-to-many’ topology, indicating that a single ancestral gene (referred to as *C*) underwent duplication following the emergence of the Rosaceae family, giving rise to the *C* and *D* genes. Subsequently, in *Prunus* species, both genes experienced additional tandem duplications and gene losses. These events led to the formation of the current gene cluster observed across various *Prunus* species, in which the original gene *C* further diversified into the current *A*, *B*, and *C* genes. In apricot *PruarA*, *PruarB* and *PruarC* correspond to *ParPMC1, ParPMC2* and *ParP5*, respectively. This aligns with the frequent gene duplication and loss events reported in Rosaceae species (Jia et al., 2015). Tandem genes have a high duplication rate per generation, continually producing new paralogs and dynamic selection targets (Hanada et al., 2008), which typically undergo pseudogenization, subfunctionalization, or neofunctionalization, though these processes can overlap (Panchy et al., 2016). As previously observed, the ParPMC1 and ParPMC2 proteins are located in distinct cellular compartments, which could suggest functional differences or the acquisition of a novel targeting sequence, allowing them to perform the ancestral function in a new subcellular compartment. Further studies are needed to clarify the role of the different members of the cluster. Interestingly, tandem duplication has been recognized as a key mechanism in the expansion of NLR-encoding genes in Rosaceae (Jia et al., 2015) and has also been identified as a significant factor enabling plants to adapt to changing environments (Das Laha et al., 2020; Yu et al., 2015). Tandem duplications have expanded orthologous groups linked to biotic stimulus response, defense, toxin response, transport functions, glycosinolate metabolism, phosphorylation, extracellular and cell surface components (Hanada et al., 2008). Moreover, tandem duplication of genes in an orthologous group is typically asymmetric, indicating lineage-specific selection pressures (Hanada et al., 2008). In this sense, future studies should explore the potential relationship between the host-strain specificity in PPV infections in *Prunus* spp. and the differences in the tandemly arrayed gene cluster among species.

### 4.3 The *ParPMC* ortholog in *N. benthamiana*, *NbPMC*, is a susceptible gene involved in PPV resistance

The identification of a *ParPMC* ortholog in *N. benthamiana* allowed us to use this heterologous system for functional assays as an easier handling alternative to the challenging genetic transformation in *Prunus*. In this work, TRV-based VIGS successfully silenced *NbPMC* expression in *N. benthamiana*, leading to a significant reduction in PPV accumulation in both local and systemic leaves. Although partial silencing by VIGS may leave residual gene expression and produce milder phenotypes than a full knock-out, our results demonstrate that it remains a valuable tool for gaining insights into gene function. Although the reduction in PPV levels is not entirely dose-dependent on *NbPMC*, indicating the potential involvement of other genetic factors in the viral replication in *N. benthamiana*, this work strongly supports the involvement of *NbPMC* as a host susceptibility factor in PPV infection. Notably, *NbPMC* silencing did not result in observable differences in plant growth compared to control plants, although growth was not quantitatively assessed in this study. Similarly, resistant apricot cultivars, which exhibit downregulation of *ParPMC1* and *ParPMC2*, show no developmental differences compared to susceptible ones.

Viral infection involves replication after entry into plant cells, followed by export to neighboring cells and entry into vascular tissue for long-distance movement, overcoming the physical and cellular barriers of the phloem (Navarro et al., 2019). Potyviral RNA replication and movement are thought to be closely linked processes, although detailed information regarding PPV remains limited (Rodamilans et al., 2020). In this study, we observed a reduction in viral load when *NbPMC* was partially silenced, as well as a more restricted or delayed virus distribution. In *NbPMC*-silenced plants, PPV spread gradually through class I, II, and III veins, whereas in control plants, a broader distribution occurred more rapidly. This may be due to a reduced viral titer, impaired cell-to-cell movement through the mesophyll, or a combination of both factors. Further research is needed to clarify which process is being affected. Overall, the results presented here support the initial hypothesis that *ParPMC1* and/or *ParPMC2* are host susceptibility genes in apricot (Zuriaga et al., 2018). Notably, our findings show that these genes are expressed in conductive tissues, suggesting a potential role in the long-distance movement of the virus. In light of the results presented in this work and considering the role of the MATHd-only proteins MUSE13 and MUSE14 in the ubiquitination-mediated modulation of plant immunity (Huang et al., 2016), we speculate that ParPMC proteins may be involved in the post-translational modifications of viral and/or host proteins necessary for viral movement. In the absence of these proteins, the virus may be unable to move efficiently, thereby impairing its systemic spread and reducing the viral load available to infect new cells. We are currently investigating the protein interactors of ParPMC1 and ParPMC2, which could provide additional support for validating this hypothesis. Furthermore, the orthology analysis opens exciting avenues for future research. It reveals that an ancestral gene underwent duplication within the Rosaceae family and later diversified within the *Prunus* genus. Since *NbPMC*—the ortholog in *N. benthamiana*—is still involved in PPV infection, the potential role of *ParP5*, in addition to *ParPMC1* and *ParPMC2*, in apricot PPV resistance cannot be ruled out. The specific functions and mechanisms of these genes in apricot viral susceptibility remain unclear and require further study—ideally in apricot to address interspecies differences—although the results from this work demonstrate that *N. benthamiana* could be a useful heterologous system in this context. This work represents an important initial step toward that goal. Ongoing studies also aim to explore whether orthologs in other species can confer resistance to different potyviruses. This work advances our understanding of the role of *ParPMC* genes in PPV resistance and further enriches the knowledge of potyvirus infection in plants.

## Data Availability

The datasets presented in this study can be found in online repositories. The names of the repository/repositories and accession number(s) can be found in the article/**Supplementary Material**.
