# Supplementary material for: ParPMC-mediated susceptibility to plum pox virus: vascular expression in Prunus armeniaca and functional validation through ortholog silencing in Nicotiana benthamiana
Source: Front Plant Sci. 2025 Jun 25;16:1614211. doi: 10.3389/fpls.2025.1614211 (PMC12238093; doi:10.3389/fpls.2025.1614211)

Supplementary Figure 3. RFP-labelled PPV spread at 4, 5 and 6 dpi in wild-type *N. benthamiana* leaves from TRV-VIGS NbPMC-silenced, GFP:NbPMC-silenced, and control plants. RFP fluorescence was observed using a Leica MZ16F stereomicroscope.

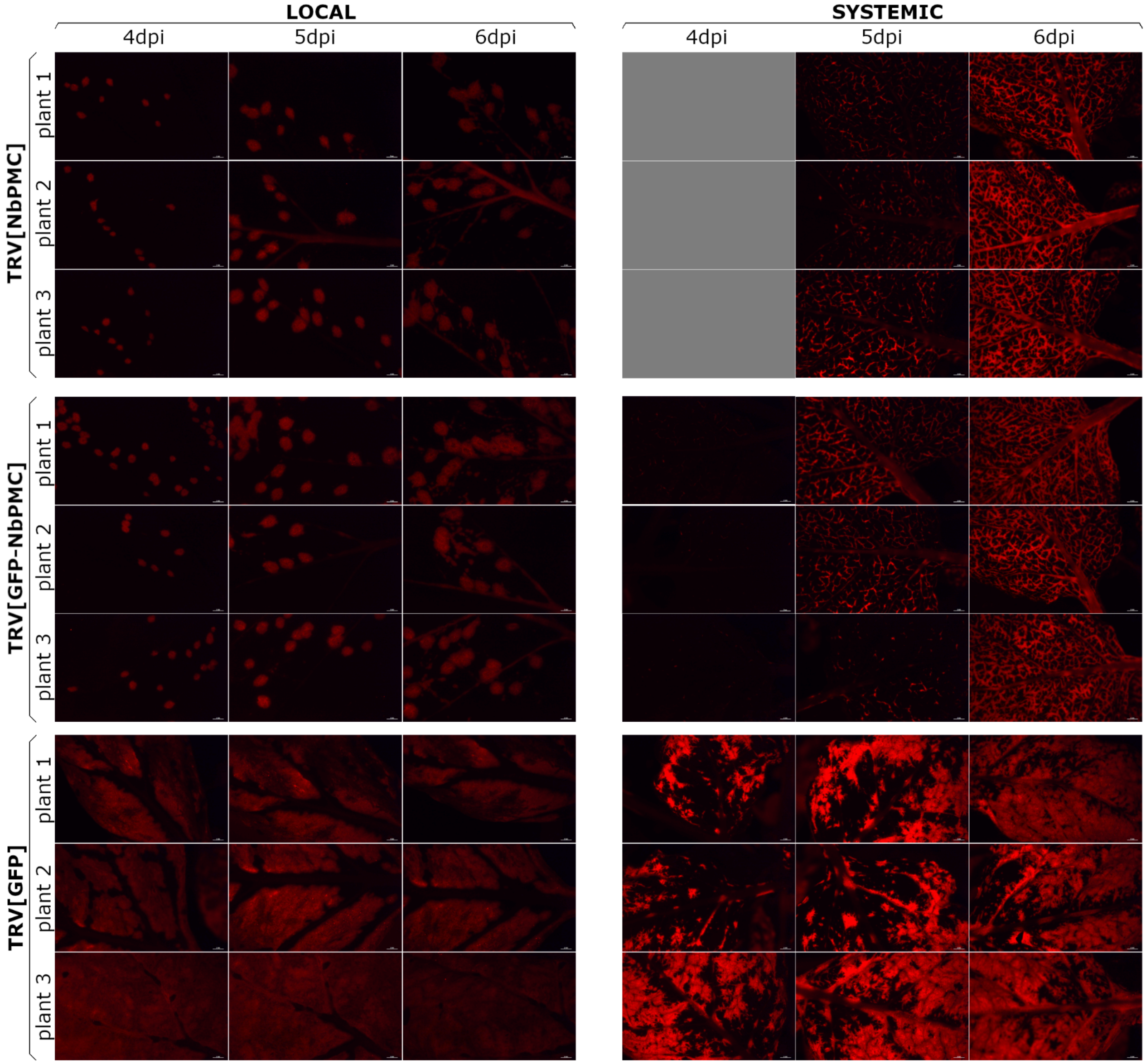

Supplement: Supplementary file 1 [file DataSheet1.zip › Supplementary_Figure_3.pdf]
