# Supplementary material for: ParPMC-mediated susceptibility to plum pox virus: vascular expression in Prunus armeniaca and functional validation through ortholog silencing in Nicotiana benthamiana
Source: Front Plant Sci. 2025 Jun 25;16:1614211. doi: 10.3389/fpls.2025.1614211 (PMC12238093; doi:10.3389/fpls.2025.1614211)
Supplement: Supplementary file 1 [file DataSheet1.zip › Supplementary_Table_1.pdf]

Supplementary Material

Supplementary Table 1. Primers used in this study.

**Experiment**	**Primer name**	**Forward**	**Reverse**
apricot qPCR	actin	CTTCTTACTGAGGCACCCCTGAAT	AGCATAGAGGGAGAGAACTGCTTG
	sand-like	TCGTGGGTACCAGGAAAACGACAT	CCTGCTAGCTTGTGTTCATCTCCA
	ParPMC1	AGAATACTGGCACGTACTTTGCTC	GTCAGTAAAAGCTTTGAGGGAGAG
	ParPMC2	TGCCAACTCATTACACGTTCA	GTGCTCTTTCACATTCTTGCTC
	ParP-5	TCTTCCTCCAGGCTCTAAAATCTAT	AAATCCTAGCGTTATGAATCTCCAC
in situ hybridization	ParPMC1_probe	GTATACCCTGCGCCTCCTAAAT	AAGGCCAGGAGAAGTGATACAA
	ParPMC2_probe	TAAGAGCTTGGGGTATTTGGAG	GGACCAAGAAGCCTTTCAGAAT
subcellular localization	ParPMC1_FP	AGGATCGGTACCATGAACATGACTAGT	CTGCACCGGCGCTAGCCTCTAGCGCACTAGCAGTT
	ParPMC2_FP	AGGATCGGTACCATGAGCATGAATAACCTTAACTTCG	CTGCACCGGCGCTAGCGTCTAGCGCACTAGCAGTTC
VIGS	attB-NbPMC	GGGGACAAGTTTGTACAAAAAAGCAGGCTTCTCCCGATCTTACAACGAAGG	GGGGACCACTTTGTACAAGAAAGCTGGGTGCTTGTCGATCTTCCGGGC
	GFP-NbPMC	GAGAGGACCATCTTCTTCAAGGTCCCGATCTTACAACGAAGG	
	attB-GFP	GGGGACAAGTTTGTACAAAAAAGCAGGCTTCAGTAAAGGAGAAGAACTTTTC	GGGGACCACTTTGTACAAGAAAGCTGGGTGTTTGTATAGTTCATCCATGCC
	PP2A	GACCCTGATGTTGATGTTCG	GAGGGATTTGAAGAGAGATTTC
	NbPMC_qPCR	GGCGGCTACAAATGGAAGTT	AGCTTCCCTCCGCCATAATC
	PPV_qPCR	CAGACTACAGCCTCGCCAGA	ACCGAGACCACTACACTCCC
