# Supplementary material for: ParPMC-mediated susceptibility to plum pox virus: vascular expression in Prunus armeniaca and functional validation through ortholog silencing in Nicotiana benthamiana
Source: Front Plant Sci. 2025 Jun 25;16:1614211. doi: 10.3389/fpls.2025.1614211 (PMC12238093; doi:10.3389/fpls.2025.1614211)
Supplement: Supplementary file 1 [file DataSheet1.zip › Supplementary_Table_3.pdf]

Supplementary Table 3. RT-qPCR analysis of *ParPMC1*, *ParPMC2* and *ParP5* genes in leaf blade (L), petiole (P), primary (V1) and secondary veins (V2). Different letters denote significant differences (P<0.05).

			ParPMC1					ParPMC2					ParP-5				
Tissue	Phenotype	Sample	Normalized Target Value	SD	Statistical difference	Relative Expression	Mean	Normalized Target Value	SD	Statistical difference	Relative Expression	Mean	Normalized Target Value	SD	Statistical difference	Relative Expression	Mean
L	R	GO	0,121	0,001	d	1,000		0,129	0,001	f	1,000		0,228	0,018	e	1,000	
		HC	1,347	0,005	bc	11,104		0,160	0,000	ef	1,241		0,521	0,051	c	2,288	
		HL	0,344	0,004	c	2,838	0,385 a	0,114	0,000	f	0,884	0,160 a	0,066	0,021	f	0,289	0,287 a
		LI	0,064	0,001	de	0,531		0,165	0,001	f	1,278		0,312	0,060	d	1,369	
		VE	0,045	0,000	e	0,372		0,229	0,002	ef	1,769		0,308	0,033	d	1,350	
	S	CA	0,783	0,008	bc	6,454		1,488	0,016	b	11,506		0,678	0,073	b	2,977	
		GI	0,612	0,007	bc	5,045		0,973	0,003	c	7,524		0,755	0,134	b	3,315	
		KA	6,476	0,074	a	53,367	1,684 a	4,946	0,013	a	38,253	1,679 b	1,981	0,035	a	8,694	0,836 b
		MI	0,367	0,001	c	3,025		0,436	0,001	d	3,373		0,385	0,137	cd	1,690	
		TA	0,184	0,001	cd	1,518		0,554	0,003	de	4,287		0,379	0,083	d	1,664	
P	R	GO	0,927	0,005	e	1,000		0,886	0,005	d	1,000		0,917	0,045	d	1,000	
		HC	0,003	0,000	g	0,003		1,265	0,017	c	1,428		0,875	0,034	de	0,953	
		HL	0,004	0,000	g	0,005	0,191 a	0,733	0,015	d	0,828	1,111 a	0,422	0,068	h	0,460	0,820 a
		LI	0,008	0,001	g	0,009		1,382	0,009	c	1,560		1,099	0,059	c	1,198	
		VE	0,012	0,000	g	0,013		1,290	0,015	c	1,457		0,785	0,067	f	0,856	
	S	CA	2,130	0,036	c	2,297		5,383	0,047	a	6,079		2,897	0,123	a	3,158	
		GI	4,515	0,088	bc	4,869		3,270	0,042	b	3,692		2,305	0,556	b	2,513	
		KA	12,631	0,165	a	13,621	4,274 b	4,601	0,078	a	5,196	3,475 b	2,406	0,213	b	2,623	1,804 a
		MI	1,580	0,054	d	1,704		2,144	0,087	b	2,421		0,821	0,153	ef	0,895	
		TA	0,515	0,010	f	0,556		1,977	0,028	b	2,233		0,592	0,076	g	0,646	
V1	R	GO	1,211	0,025	d	1,000		0,980	0,008	ef	1,000		1,416	0,256	de	1,000	
		HC	0,071	0,001	g	0,059		1,254	0,004	d	1,279		1,393	0,077	e	0,984	
		HL	0,234	0,003	e	0,193	0,354 a	0,721	0,005	f	0,735	1,019 a	0,592	0,074	g	0,418	1,090 a
		LI	0,151	0,003	f	0,125		1,106	0,014	ef	1,128		1,053	0,073	f	0,744	
		VE	0,102	0,001	f	0,084		1,033	0,005	ef	1,053		0,997	0,083	f	0,705	
	S	CA	0,969	0,009	d	0,800		3,667	0,030	b	3,740		1,671	0,235	cd	1,180	
		GI	2,984	0,061	bc	2,465		3,681	0,090	ab	3,754		3,651	0,666	a	2,579	
		KA	3,492	0,021	a	2,884	2,016 b	2,609	0,020	c	2,661	3,517 b	2,725	0,210	b	1,925	2,285 a
		MI	1,757	0,015	c	1,451		3,488	0,014	b	3,557		1,715	0,088	c	1,212	
		TA	0,880	0,026	d	0,727		4,143	0,007	a	4,225		1,661	0,083	c	1,173	
V2	R	GO	0,328	0,006	d	1,000		0,510	0,006	f	1,000		0,639	0,043	e	1,000	
		HC	0,108	0,001	e	0,328		0,927	0,008	d	1,817		1,024	0,162	cd	1,602	
		HL	0,048	0,002	f	0,147	0,168 a	0,584	0,003	f	1,145	0,717 a	0,495	0,047	f	0,774	0,778 a
		LI	0,246	0,001	d	0,749		0,801	0,001	d	1,569		0,959	0,037	c	1,500	
		VE	0,113	0,002	ef	0,343		0,761	0,004	d	1,492		0,775	0,131	d	1,213	
	S	CA	1,611	0,008	bc	4,911		4,508	0,005	a	8,834		2,134	0,079	b	3,339	
		GI	0,269	0,010	de	0,820		0,263	0,026	g	0,515		0,166	0,208	g	0,261	
		KA	4,475	0,057	a	13,644	1,749 b	3,074	0,013	b	6,023	2,834 b	2,274	0,175	b	3,558	1,656 a
		MI	1,251	0,009	c	3,814		1,571	0,003	c	3,078		0,951	0,051	c	1,488	
		TA	1,142	0,005	c	3,481		4,756	0,003	a	9,319		2,753	0,101	a	4,309	
