# Supplementary material for: ParPMC-mediated susceptibility to plum pox virus: vascular expression in Prunus armeniaca and functional validation through ortholog silencing in Nicotiana benthamiana
Source: Front Plant Sci. 2025 Jun 25;16:1614211. doi: 10.3389/fpls.2025.1614211 (PMC12238093; doi:10.3389/fpls.2025.1614211)
Supplement: Supplementary file 1 [file DataSheet1.zip › Supplementary_Table_4.pdf]

Table S4. List of species used for the orthology analysis, with links to their respective genome databases (all accessed 23/12/2024).

N.	Family	Subfamily	Spp	Genome version	Genome website
1	Rosaceae	Amygdaloideae	**Prunus armeniaca**	Prunus armeniaca cv.Stella Whole Genome v1.0 Assembly & Annotation	https://www.rosaceae.org/Analysis/11326140
	Rosaceae	Amygdaloideae	**Prunus armeniaca**	Prunus armeniaca Longwangmao Whole Genome v1.0 Assembly & Annotation	https://www.rosaceae.org/Analysis/10254126
	Rosaceae	Amygdaloideae	**Prunus armeniaca**	Prunus armeniaca Marouch n14 Whole Genome v1.0 Assembly & Annotation	https://www.rosaceae.org/Analysis/9642068
	Rosaceae	Amygdaloideae	**Prunus armeniaca**	Prunus armeniaca Sungold Whole Genome v1.0 Assembly & Annotation	https://www.rosaceae.org/Analysis/10254125
	Rosaceae	Amygdaloideae	**Prunus armeniaca (RNAseq)**	Transcriptome profiling of apricot (Prunus armeniaca) cultivars in response to Plum Pox Virus (PPV) infection (Zuriaga et al., 2018)	https://www.ncbi.nlm.nih.gov/bioproject/PRJNA387702/
2	Rosaceae	Amygdaloideae	**Prunus avium**	Prunus avium Regina Genome v1.0 Assembly & Annotation	https://www.rosaceae.org/Analysis/17988772
3	Rosaceae	Amygdaloideae	**Prunus cerasus**	Prunus cerasus cv. 'Montmorency' Whole Genome v1.0.a2 Assembly & Annotation	https://www.rosaceae.org/Analysis/18356082
4	Rosaceae	Amygdaloideae	**Prunus domestica**	Prunus domestica Draft Genome Assembly v1.0 & Annotation v1.0.a1	https://www.rosaceae.org/species/prunus_domestica/genome_v1.0.a1
5	Rosaceae	Amygdaloideae	**Prunus dulcis**	Prunus dulcis Texas Genome v2.0 (Alioto et al., 2020)	https://www.rosaceae.org/analysis/295
6	Rosaceae	Amygdaloideae	**Prunus fruticosa**	Prunus fruticosa 27e12(2) Whole Genome v1.0 Assembly & Annotation	https://www.rosaceae.org/Analysis/12335063
7	Rosaceae	Amygdaloideae	**Prunus humilis**	Prunus humilis Genome v1.0 Assembly & Annotation	https://www.rosaceae.org/Analysis/13738172
8	Rosaceae	Amygdaloideae	**Prunus kansuensis**	Prunus kansuensis Whole Genome v2.0 Assembly & Annotation	https://www.rosaceae.org/Analysis/12080706
9	Rosaceae	Amygdaloideae	**Prunus mandshurica**	Prunus mandshurica CH264_4 Whole Genome v1.0 Assembly & Annotation	https://www.rosaceae.org/Analysis/10024324
10	Rosaceae	Amygdaloideae	**Prunus mira**	Prunus mira Whole Genome v2.0 Assembly & Annotation (Cao et al., 2022)	https://www.rosaceae.org/Analysis/12080707
11	Rosaceae	Amygdaloideae	**Prunus mongolica**	Prunus mongolica Whole Genome v1.0 Assembly & Annotation	https://www.rosaceae.org/Analysis/16296039
12	Rosaceae	Amygdaloideae	**Prunus mume**	Genome assembly P.mume_V1.0	https://www.ncbi.nlm.nih.gov/datasets/genome/GCF_000346735.1/
13	Rosaceae	Amygdaloideae	**Prunus persica**	Prunus persica Whole Genome Assembly v2.0 & Annotation v2.1 (v2.0.a1)	https://www.rosaceae.org/species/prunus_persica/genome_v2.0.a1
14	Rosaceae	Amygdaloideae	**Prunus sibirica**	Prunus sibirica CH320_5 Whole Genome v1.0 Assembly & Annotation	https://www.rosaceae.org/Analysis/9955981
	Rosaceae	Amygdaloideae	**Prunus sibirica**	Prunus sibirica F106 Whole Genome v1.0 Assembly & Annotation	https://www.rosaceae.org/Analysis/10254124
15	Rosaceae	Pyroideae	**Malus x domestica**	Genome assembly ASM211411v1	https://www.ncbi.nlm.nih.gov/datasets/genome/GCF_002114115.1/
16	Rosaceae	Pyroideae	**Pyrus bretschneideri**	Genome assembly Pyrus_bretschneideri_v1 (2021)	https://www.ncbi.nlm.nih.gov/datasets/genome/GCF_019419815.1/
17	Rosaceae	Pyroideae	**Pyrus communis**	Pyrus communis Bartlett DH Genome v2.0	https://www.rosaceae.org/species/pyrus/pyrus_communis/genome_v2.0
18	Rosaceae	Rosoideae	**Fragaria vesca**	Fragaria vesca Whole Genome v4.0.a2 (Re-annotation of v4.0.a1)	https://www.rosaceae.org/species/fragaria_vesca/genome_v4.0.a2
19	Rosaceae	Rosoideae	**Rosa chinensis**	Rosa chinensis Old Blush homozygous genome v2.0	https://www.rosaceae.org/analysis/282
20	Brassicaceae	Brassicoideae	**Arabidopsis thaliana**	TAIR 10	https://ftp.ncbi.nlm.nih.gov/genomes/all/GCF/000/001/735/ GCF_000001735.3_TAIR10/
21	Brassicaceae	Brassicoideae	**Brassica oleracea**	Genome assembly BOL (2014)	https://www.ncbi.nlm.nih.gov/datasets/genome/GCF_000695525.1/
22	Caricaceae		**Carica papaya**	Genome assembly Papaya1.0	https://www.ncbi.nlm.nih.gov/datasets/genome/GCF_000150535.2/
23	Cucurbitaceae	Cucurbitoideae	**Cucumis melo**	Melonv4, INSDC Assembly GCA_902497455.1,	https://plants.ensembl.org/Cucumis_melo/Info/Annotation/
24	Cucurbitaceae	Cucurbitoideae	**Cucurbita pepo**	Cucurbita pepo (Zucchini) Genome v.4.1	ftp://cucurbitgenomics.org/pub/cucurbit/genome/Cucurbita_pepo/
25	Fabaceae	Faboideae	**Medicago truncatula**	MedtrA17_4.0, INSDC Assembly GCA_000219495.2, Jun 2014	https://www.ncbi.nlm.nih.gov/datasets/genome/GCF_000219495.2/
26	Fabaceae	Faboideae	**Phaseolus vulgaris**	Phaseolus vulgaris v2.1	https://phytozome.jgi.doe.gov/pz/portal.html#!info?alias=Org_Pvulgaris
27	Malvaceae	Byttnerioideae	**Theobroma cacao**	Genome assembly Criollo_cocoa_genome_V2 (2016)	https://www.ncbi.nlm.nih.gov/datasets/genome/GCF_000208745.1/
28	Salicaceae	Salicoideae	**Populus trichocarpa**	Genome assembly P.trichocarpa_v4 (2022)	https://www.ebi.ac.uk/ena/browser/view/GCA_000002775.4
29	Solanaceae	Nicotianoideae	**Nicotiana benthamiana**	Nicotiana benthamiana draft genome sequence v2.6.1	https://solgenomics.net/organism/Nicotiana_benthamiana/genome
30	Solanaceae	Solanoideae	**Solanum melongena**	Eggplant genome consortium V4.1	https://solgenomics.net/organism/Solanum_melongena/genome
31	Solanaceae	Solanoideae	**Solanum tuberosum**	S. tuberosum Group Phureja DM1-3 516R44 (CIP801092) Genome Annotation v3.4	https://solgenomics.net/organism/Solanum_tuberosum/genome
