# Supplementary material for: ParPMC-mediated susceptibility to plum pox virus: vascular expression in Prunus armeniaca and functional validation through ortholog silencing in Nicotiana benthamiana
Source: Front Plant Sci. 2025 Jun 25;16:1614211. doi: 10.3389/fpls.2025.1614211 (PMC12238093; doi:10.3389/fpls.2025.1614211)
Supplement: Supplementary file 1 [file DataSheet1.zip › Supplementary_Table_5.pdf]

Supplementary Table 5. Estimates of average evolutionary divergence over sequence pairs within groups, using the JTT+G model (shape parameter = 5), with 124 sequences. The number of amino acid substitutions per site from averaging over all sequence pairs within each group are shown. All ambiguous positions were removed for each sequence pair.

Gene Group		N.	Divergence
A		18	**0.14**
B		16	**0.04**
C		32	0.38
	C_Rosaceae_Prunus	16	**0.04**
	C_Rosaceae_non_Prunus	5	0.23
	C_nonRosacea	11	0.59
D		17	0.27
	D_Prunus	13	**0.09**
	D_nonPrunus	4	0.33
F		9	**0.09**
G		7	0.22
	G_Prunus	6	**0.07**
H		10	**0.05**
I		15	**0.05**

In bold groups with just *Prunus* sequences
