# Supplementary material for: ParPMC-mediated susceptibility to plum pox virus: vascular expression in Prunus armeniaca and functional validation through ortholog silencing in Nicotiana benthamiana
Source: Front Plant Sci. 2025 Jun 25;16:1614211. doi: 10.3389/fpls.2025.1614211 (PMC12238093; doi:10.3389/fpls.2025.1614211)
Supplement: Supplementary file 1 [file DataSheet1.zip › Supplementary_Table_6.pdf]

Supplementary Table 6. *NbPMC* gene expression and PPV accumulation analyzed by RT-qPCR in inoculated (local) and non-inoculated (systemic) upper leaves of wild-type (WT) and GFP16c (constitutively expressing GFP) *N. benthamiana* plants from the TRV-VIGS experiment. Different letters denote significant differences (P<0.05).

			NbPMC				PPV			
	Ecotype	TRV	$2^{-\Delta\Delta Ct}$	$< 2^{-\Delta\Delta Ct} >$	<SD>	t-student	$2^{-\Delta\Delta Ct}$	$< 2^{-\Delta\Delta Ct} >$	<SD>	t-student
LOCAL	WT	[NbPMC]	0,316<br>0,260<br>0,355	0,310	0,047	a	0,175<br>0,215<br>0,148	0,179	0,034	a
LOCAL	WT	[GFP-NbPMC]	0,582<br>0,143<br>0,142	0,289	0,254	a	0,522<br>0,116<br>0,021	0,220	0,266	a
LOCAL	WT	[GFP]	0,815<br>1,372<br>0,895	1,027	0,301	b	0,922<br>0,909<br>1,193	1,008	0,161	b
LOCAL	GFP16c	[NbPMC]	0,203<br>0,233<br>0,331	0,255	0,067	a	0,208<br>0,136<br>0,124	0,156	0,045	a
LOCAL	GFP16c	[GFP-NbPMC]	0,357<br>0,157<br>0,196	0,237	0,106	a	0,254<br>0,163<br>0,239	0,219	0,049	a
LOCAL	GFP16c	[GFP]	1,095<br>1,158<br>0,788	1,014	0,198	b	1,022<br>1,005<br>0,974	1,000	0,024	b
SYSTEMIC	WT	[NbPMC]	0,319<br>0,240<br>0,337	0,299	0,052	a	0,320<br>0,495<br>0,412	0,409	0,087	a
SYSTEMIC	WT	[GFP-NbPMC]	0,175<br>0,356<br>0,483	0,338	0,155	a	0,461<br>0,579<br>0,537	0,526	0,060	a
SYSTEMIC	WT	[GFP]	1,273<br>1,155<br>0,680	1,036	0,314	b	0,942<br>1,089<br>0,975	1,002	0,077	b
SYSTEMIC	GFP16c	[NbPMC]	0,332<br>0,320<br>0,409	0,354	0,048	a	0,212<br>0,263<br>0,256	0,244	0,028	a
SYSTEMIC	GFP16c	[GFP-NbPMC]	0,312<br>0,196<br>0,274	0,261	0,059	a	0,466<br>0,520<br>0,707	0,564	0,126	b
SYSTEMIC	GFP16c	[GFP]	1,272<br>1,079<br>0,729	1,027	0,276	b	1,006<br>0,932<br>1,066	1,002	0,067	c
