# Supplementary material for: ParPMC-mediated susceptibility to plum pox virus: vascular expression in Prunus armeniaca and functional validation through ortholog silencing in Nicotiana benthamiana
Source: Front Plant Sci. 2025 Jun 25;16:1614211. doi: 10.3389/fpls.2025.1614211 (PMC12238093; doi:10.3389/fpls.2025.1614211)

Supplementary Figure 2. GFP16c *N. benthamiana* plants from the TRV-VIGS experiments, 6 days after agroinfiltration with pTRV1 and pTRV2[NbPMC], pTRV2[GFP:NbPMC], or pTRV2[GFP], and non-inoculated controls. Scale bar = 10 cm.

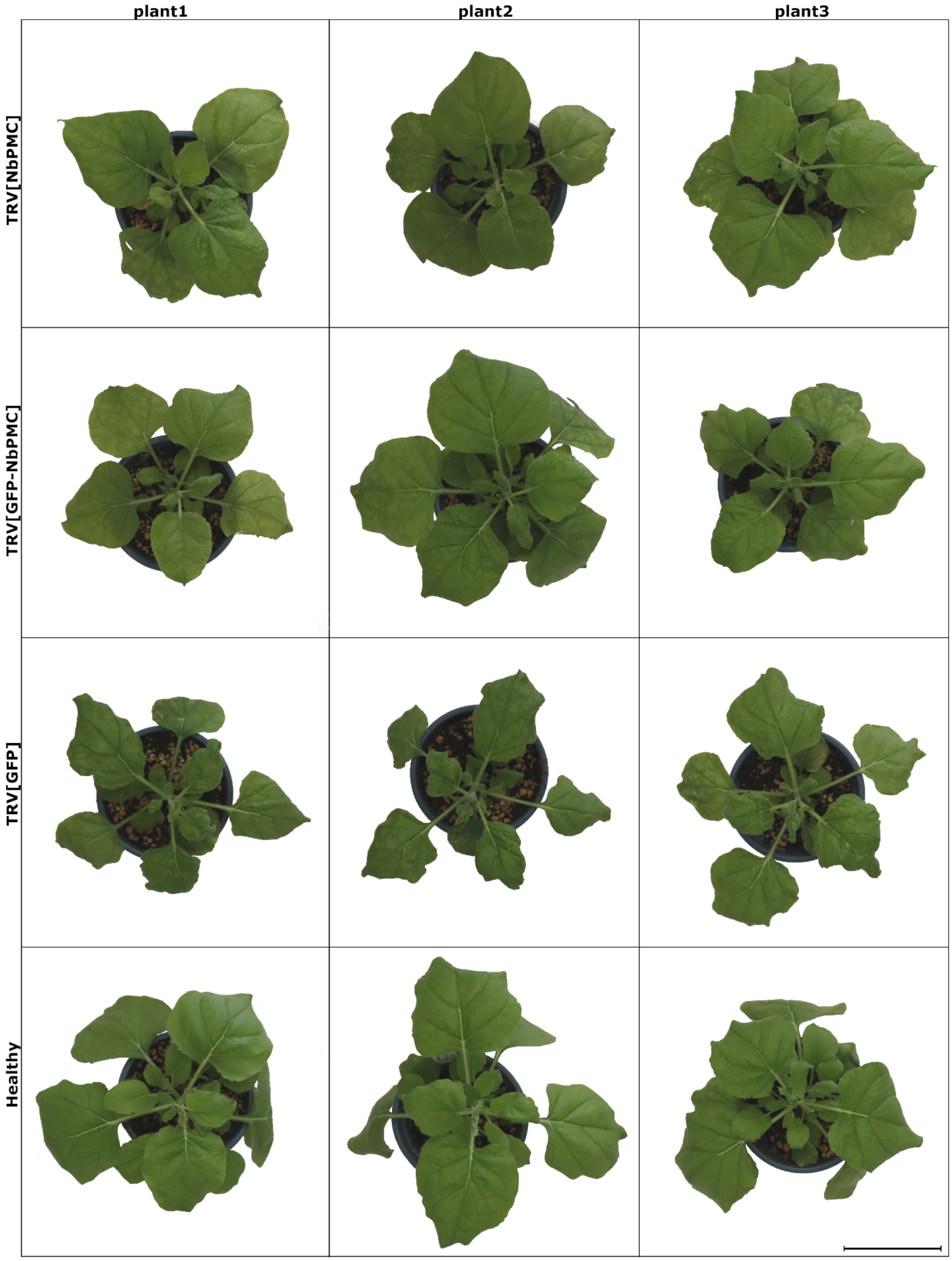

Supplement: Supplementary file 1 [file DataSheet1.zip › Supplementary_Figure_2.pdf]
